# Protein Z Exerts Pro-Angiogenic Effects and Upregulates CXCR4

**DOI:** 10.1371/journal.pone.0113554

**Published:** 2014-12-04

**Authors:** Antje Butschkau, Nana-Maria Wagner, Berit Genz, Brigitte Vollmar

**Affiliations:** 1 Institute for Experimental Surgery, University Hospital Rostock, Rostock, Germany; 2 Clinic for Anesthesiology and Critical Care Medicine, University Hospital Rostock, Rostock, Germany; University Hospital Medical Centre, Germany

## Abstract

**Objective:**

Protein Z (PZ) is a vitamin K-dependent coagulation factor without catalytic activity. Evidence points towards PZ as an independent risk factor for the occurrence of human peripheral arterial disease. However, the role of PZ in ischemia-driven angiogenesis and vascular healing processes has not been elucidated so far.

**Approach:**

Angiogenic potency of PZ was assessed in established *in vitro* assays using endothelial cells. PZ-deficient (PZ^−/−^) mice and their wild-type littermates (PZ^+/+^) were subjected to hindlimb ischemia. Furthermore, PZ^−/−^ mice were exposed to PZ expressing adenovirus (AdV-PZ) or control adenovirus (AdV-GFP). In an additional set of animals, PZ^−/−^ mice were exposed to AdV-PZ and AdV-GFP, each in combination with the CXCR4 antagonist AMD3100.

**Results:**

*In vitro*, PZ stimulated migratory activity and capillary-like tube formation of endothelial cells comparable to SDF-1. PZ^−/−^ mice exhibited diminished hypoxia-driven neovascularization and reperfusion in post-ischemic hindlimbs, which was restored by adenoviral gene transfer up to levels seen in PZ^+/+^ mice. The stimulatory impact of PZ on endothelial cells *in vitro* was abolished by siRNA targeting against PZ and PZ was not able to restore reduced migration after knock-down of CXCR4. The increased surface expression of CXCR4 on PZ-stimulated endothelial cells and the abrogated restoration of PZ^−/−^ mice via AdV-PZ after concomitant treatment with the CXCR4 antagonist AMD3100 supports the idea that PZ mediates angiogenesis via a G-protein coupled pathway and involves the SDF-1/CXCR4 axis. This is underlined by the fact that addition of the G-protein inhibitor PTX to PZ-stimulated endothelial cells abolished the effect of PZ on capillary-like tube formation.

**Conclusions:**

The results of the current study reveal a role of PZ in ischemia-induced angiogenesis, which involves a G-protein coupled pathway and a raised surface expression of CXCR4. Our findings thereby extend the involvement of PZ from the coagulation cascade to a beneficial modulation of vascular homeostasis.

## Introduction

Protein Z (PZ) is a 62 kDa vitamin K-dependent coagulation glycoprotein with a molecular structure similar to those of factor VII, IX, X and protein C [1] and a biological half-life of 2.5 days [2]. PZ was initially identified by Prowse and Esnouf in bovine plasma in 1977 [3] and the human homologue was subsequently isolated in 1984 [2]. In contrast to the serine protease zymogens, PZ lacks catalytic activity [4] and serves as a cofactor for the protein Z-dependent protease inhibitor (ZPI), a 72 kDa member of the serpin superfamily of protease inhibitors [5], [6]. PZ and ZPI deficiency have been shown to enhance thrombosis in mouse models [7], [8]. Whether PZ and ZPI are involved in clinical thrombotic disease is controversially discussed, with some, but not all, studies suggesting a relation. In humans, low PZ-levels are associated with the occurrence of deep vein thrombosis [9] and increased risk of ischemic stroke [10]. In 2007, Sofi et al. observed an association between low PZ-levels and both the occurrence and the severity of peripheral arterial disease (PAD) in a case control study, postulating evidence for a role of PZ in the pathogenesis of atherosclerotic disease [11]. In 2009 they could confirm these results by another case-control study, demonstrating again a significant association of low PZ-levels with the occurrence and severity of PAD [12].

Several studies point towards a role of coagulation factors in angiogenic processes. After first evidence for a relevance of heparin cofactor II in the development of atherosclerosis [13], Ikeda and coworkers found that heparin cofactor II potentiates the activation of vascular endothelial cells and promotes angiogenesis in response to hindlimb ischemia via an AMP-activated protein kinase-endothelial nitric-oxide synthase signaling pathway [14]. Protein C, a structural homologue to PZ, was shown to be associated with lower leg ulcers in patients with diabetes, when protein C plasma levels were diminished [15]. Already in the early 1990's, endothelial cells were identified as a source for protein C [16]. Uchiba et al. found that activated Protein C (aPC) induces endothelial cell proliferation *in vitro* and angiogenesis in mouse cornea *in vivo* [17]. Moreover, it was shown that aPC promotes the barrier function of HUVECs by utilization of the angiopoietin/Tie2 axis [18], [19].

Like protein C, PZ is expressed by endothelial cells and immunolocalized in human vessel sections, where the labeling was found stronger on arterial than venular endothelium [20]. Though reasonable, the role of PZ for angiogenesis and neovascularization has not been elucidated so far. In the present study, we investigated the modulatory capacity of PZ on ischemia-induced neovascularization employing PZ-deficient mice and explored the response of endothelial cells to stimulation with physiological concentrations of PZ *in vitro*. Moreover, we identified the receptor of the stromal cell-derived factor-1 (SDF-1) CXCR4 as being part of the molecular mechanism underlying the effects of PZ on endothelial cells.

## Materials and Methods

### Cell Culture of Endothelial Cells

HUVECs were purchased from Lonza (Basel, Switzerland) and cultivated in endothelial basal medium with supplements and growth factors (EBM-2 and EGM-2, Lonza, Basel, Switzerland) supplemented with 10% fetal calf serum on gelatin-coated dishes (Attachment Factor, Gibco, Germany). Cells were harvested by trypsinization (0.05%, Gibco, Germany) and used from passage 2 to 5.

### Matrigel Angiogenesis Assay

As described previously [21], 1×10^4^ HUVECs were incubated with PZ (3 µg/ml; Sigma Aldrich, St. Louis, MO, USA/Enzyme Research Laboratories, South Bend, IN, USA), SDF-1 (50 ng/ml; R&D Systems, Minneapolis, MN, USA; #350-NS-010) and Pertussis Toxin (PTX, 100 ng/ml; Tocris Bioscience, Bristol, UK) in duplicate in 100 µL endothelial growth medium for 8 hours in 96-well plates precoated with 70 µL Matrigel Basement Membrane Matrix (BD Bioscience, USA). Tubular HUVEC structures were photographed using a fluorescence microscope (Leica, Germany) employing 100× magnification at 8 random high power fields (HPF) per variant. Tubular length was assessed per high-power field employing ImageProPlus Software, (CA, USA). Per independent experiment, mean values of all variants were expressed as relative to control (ctrl = 1.0).

### Migration Assay

HUVECs were grown on 12-well plates until confluence. A 10 µL pipette tip was employed to scratch over well plates twice vertically and horizontally for obtaining four 90° crosses on each well. Wells were gently washed and 2 mL of medium containing PZ (3 µg/ml), SDF-1 (50 ng/ml) or vehicle (aqueous glycerol solution) were added in triplicate per variant. All scratched-crosses were photographed every other hour until a total of 24 hours of incubation and scratch wounds were analyzed employing ImageProPlus Software. Per independent experiment, mean values of all variants were expressed as relative to control (ctrl = 1.0).

### Flow cytometry analysis

Following incubation with PZ (3 µg/ml) and vehicle, HUVECs were detached, washed and resuspended in 0.5% BSA in PBS in a concentration of 1×10^6^ cells per ml. A volume of 100 µl cell suspension was incubated with 10 µl of PE-conjugated monoclonal antibody against human CXCR4 or control IgG (both R&D Systems) and analyzed employing a BD Biosciences Becton Dickinson flow cytometer FACSCalibur (Heidelberg, Germany) after 8 and 24 hours of stimulation.

### Knock-down of CXCR4 and PZ in HUVECs

For knock-down of CXCR4- and PZ, 70–80% confluent HUVECs in a 6-well or 12-well plate were transfected with CXCR4 siRNA (sc-35421, Santa Cruz Biotechnology, USA) or PZ siRNA (sc-106450, Santa Cruz Biotechnology, USA) employing lipofectamine (Invitrogen). Cells were transfected under antibiotic-free and serum-reduced conditions for 6 hours. Then, medium was changed and cells were incubated for another 24 hours according to the manufactur's instructions. In the following, cells were used for employing the migration assay and matrigel angiogenesis assay. HUVECs transfected with ctrl siRNA (sc-37007, Santa Cruz Biotechnology, USA) and un-transfected cells served as controls.

### Immunofluorescence staining and confocal laser scanning microscopy

HUVECs were seeded in gelatin-coated 8 well μ-slides (ibidi GmbH, Martinsried, Germany) at a density of 5×10^3^ cells per well. Cells were serum-starved for 24 hours before stimulation with PZ (3 µg/ml) or SDF-1 (50 ng/ml) in serum- and growth-factor reduced medium (containing 5% FCS). After 8 and 24 hours of stimulation cells were fixed with 4% paraformaldehyde, permeabilized with 0.1% Triton-X-100 and blocked with 1% bovine serum albumin in PBS for 1 hour. Immunofluorescence staining was performed with primary antibody anti-CXCR4 (1∶50, MAB21651, R&D Systems) over night at 4°C followed by incubation with secondary antibody goat-anti-rat-Alexa^555^ (1∶400, Life Technologies GmbH, Darmstadt, Germany) for 1 hour at room temperature. Samples without primary antibodies served as negative control. Additionally, nuclei were stained with DAPI (1∶1000; AppliChem, Darmstadt, Germany) for 10 min at room temperature. The fluorescence signals were visualized by using a confocal laser scanning microscope (LSM 780 ELYRA PS.1 microscope, Carl Zeiss Microscopy GmbH, Jena, Germany).

### Mice

The experiments were conducted in accordance with the guidelines for the Care and Use of Laboratory Animals and the Institutional Animal Care and Use Committee (University of Rostock, Medical Faculty, Rostock, Germany; 7221.3-1-055/13). PZ-deficient mice (PZ^−/−^) in a C57Bl/6×129 genetic background, as described by Yin et al. and Zhang et al., were compared to their respective wild-type littermates (PZ^+/+^). Representative image and method of PCR for genotyping of PZ mice can be found in (Figure S1). Male mice were used at an age of 2–4 months and a body weight of 25–30 g. Animals were kept on water and standard laboratory chow ad libitum.

### Ethic statement

All experiments were approved by the local government (Landesamt für Landwirtschaft, Lebensmittelsicherheit und Fischerei Mecklenburg-Vorpommern LALLF M-V/TSD/7221.3-1-055/13-1) and performed in accordance with the German legislation on protection of animals and the National Institutes of *“Health Guide for the “Health Guide for the Care and Use of Laboratory Animals”* (Institute of Laboratory Animal Resources, National Research Council; NIH publication 86-23 revised 1985).

### Experimental groups

To verify the contribution of PZ in murine hindlimb ischemia, PZ^+/+^ and PZ^−/−^ mice were used. Subsequently, PZ expressing adenoviral vector (AdV-PZ) or the only GFP expressing control adenoviral vector (AdV-GFP; which was provided by B. M. Pützer; Institue for Experimental Gene Therapy and Cancer Research, University Hospital Rostock) were applicated to PZ^−/−^ mice by intravenous injection (1*10^8^ particles in saline) one day before and every seventh day after preparation of hindlimb ischemia. Constant expression and secretion of PZ over 96 hours into blood was ensured by an ELISA for PZ (for further details of adenovirus vector production, please see Material and Methods S1 and for *in vitro* and *in vivo* kinetics of adenovirus see Figure S2 and S3). To address the possible interaction of PZ with CXCR4, PZ^−/−^ mice were exposed to AdV-PZ or AdV-GFP in combination with three single injections of AMD3100 (5 mg/kg bw; subcuteanous; AMD3100 octahydrocloride hydrate, Sigma Aldrich, St. Louis, MO, USA) on post-operative day (POD) one, three and five.

### Murine hindlimb ischemia model

Unilateral hindlimb ischemia was induced as previously described [21], [22]. Mice were anesthetized by an intraperitoneal injection of ketamine (75 mg/kg bw) and xylazine (5 mg/kg bw) and placed on a warming pad to maintain the body temperature at 37°C. Then, the right femoral artery (immediately distal to the branch of the deep femoral artery) as well as the distal portion of the saphenous artery were permanently ligated employing a 7-0 polypropylene suture (Prolene, Ethicon, Norderstedt, Germany) and the ligated femoral artery was removed. Wounds were carefully sutured using 6-0 sutures (Prolene).

### Infrared thermal imaging (thermography)

Thermal imaging was performed as previously described [23]. In brief, before and immediately after surgical ligation of the femoral artery and during follow-up on post-operative day (POD) 21, mice were anesthetized as described above and placed on a 37°C heating pad for 6 min following 3 min on a table surface at room temperature and infrared imaging was performed employing a Therma CAM B20HS camera (FLIR Systems, Wilsonville, OR, USA). Images were analyzed using FLIR QuickReport 1.2 software by determination of the temperature at the middle of the pad of both the operated and non-operated hindlimb.

### Immunohistochemistry

Prior to harvest of M. gastrocnemius muscle tissue on POD 21, 100 µl of fluorescein griffonia (bandeiraea) simplicifolia lectin I (Vector Laboratories, Burlingame, CA, USA) were applied by left ventricular injection in anesthetized mice for visualization of perfused tissues. Ten min later, mice were euthanized and capillary density in the gastrocnemius muscle was assessed on 6 µm thick, acetone-fixed frozen sections after staining with antibody against CD31 (1∶50 dilution; Santa Cruz, USA) followed by Alexa555-labeled secondary antibody (Molecular Probes, USA). Cell nuclei were counterstained with DAPI. The number of CD31-immunopositive cells per muscle fiber was manually counted on 7 random microscope fields per section (200× magnification).

### Statistical analysis

All data are given as median and interquartile range (IQR; the 25% and 75% percentiles). Differences between more than two groups were calculated using ANOVA on ranks, followed by the appropriate post-hoc comparison test and between two groups using Mann-Whitney rank-sum test. Overall statistical significance was set at p<0.05 and Bonferroni corrected for Mann-Whitney rank-sum test. The statistical power was calculated for each significance at α = 0.05 with a level of 80%. Statistics, power calculation and graphics were performed using the software packages SigmaStat software version 3.5 and SigmaPlot software version 12.5 (Jandel Corporation, San Rafael, CA, USA).

## Results

### PZ promotes the angiogenic potency of endothelial cells *in vitro*

**Figure 1:**
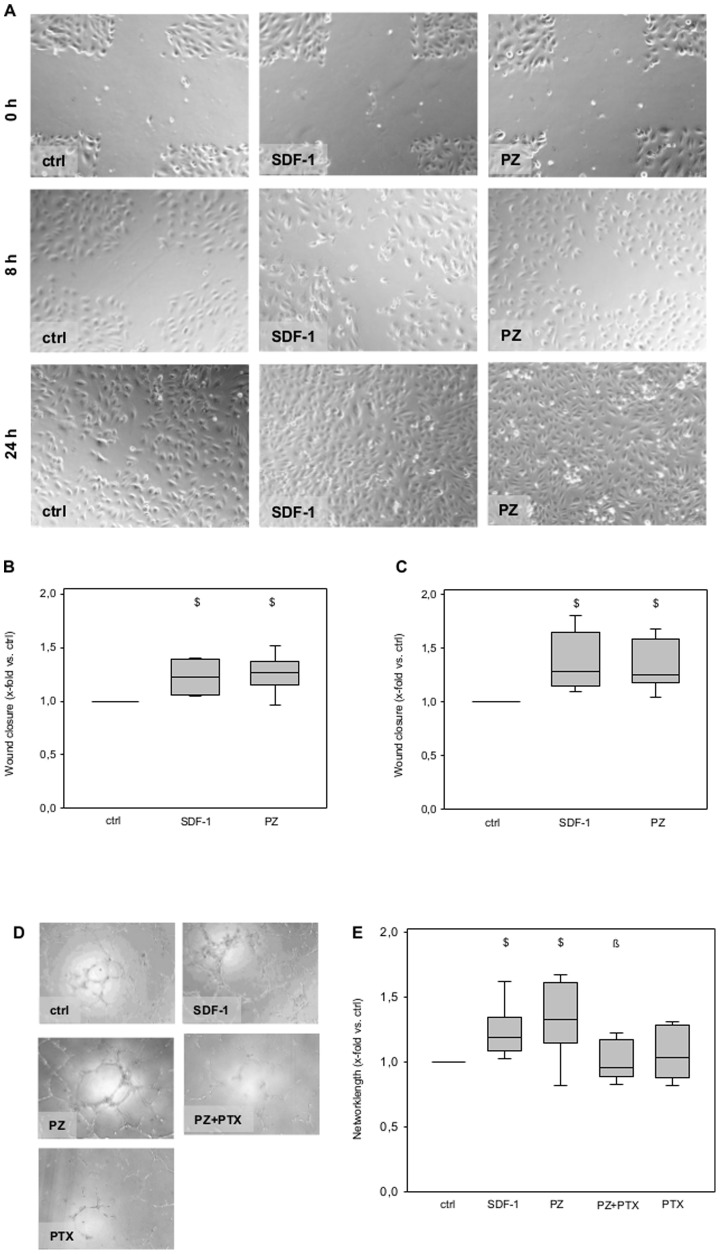
PZ promotes the migration and capillary-like tube formation of endothelial cells *in vitro*. **A**, Representative images of scratch-wound closures after 8 and 24 hours of incubation with SDF-1 or PZ, ctrl corresponds to untreated cells. 100-fold magnification. Exposure of endothelial cells to PZ (3 µg/ml) led to a significantly higher wound closure after 8 (**B**) and 24 hours (**C**) comparable to stimulation with SDF-1 (50 ng/ml). Data are given as box plots indicating the median with the 25^th^ and 75^th^ percentiles. ANOVA on ranks; $ p<0.05 vs. ctrl; n = 4–7 independent experiments. **D**, Representative images of tubular networks after 8 hours of incubation in matrigel angiogenesis assay in the presence of SDF-1 (50 ng/ml), PZ (3 µg/ml), PZ+PTX and PTX alone (100 ng/ml), ctrl are untreated cells. 100-fold magnification. **E**, HUVECs showed significantly enhanced formation of capillary-like tubular structures on Matrigel when incubated with SDF-1 and PZ compared with ctrl. Coincubation of endothelial cells with PZ and PTX abolished the PZ-mediated increase in tube formation, while incubation with PTX alone had no effect on tube formation. Data are given as box plots indicating the median with the 25^th^ and 75^th^ percentiles. ANOVA on ranks; $ p<0.05 vs. ctrl; ß p<0.05 vs. PZ; n = 6 independent experiments.

In the process of new blood vessel formation (angiogenesis), endothelial cell migration and the formation of early capillaries are prerequisites [24]. We investigated the effect of PZ on the migratory capacity of endothelial cells employing a scratch-wound assay of confluent monolayers. In the presence of PZ in a physiological concentration (3 µg/ml) endothelial cells exhibited an significantly enhanced potency to close scratch wounds by endothelial migration within 8 and 24 hours of incubation, compared with unstimulated cells (ctrl; Figure 1 A–C). This promotion of endothelial cell migration by PZ was comparable with that induced by 50 ng/ml SDF-1 (serving as positive control). Furthermore, we investigated the capacity of PZ to modulate endothelial tube formation on matrigel compared with SDF-1. After 8 hours of incubation, endothelial cells had formed significantly increased lengths of tubular networks in response to incubation with SDF-1 and PZ compared with untreated control (Figure 1 D and E). The effect on tube formation of PZ was comparable in magnitude with that exerted by SDF-1.

### PZ deficiency causes decreased ischemia-induced neovascularization

**Figure 2:**
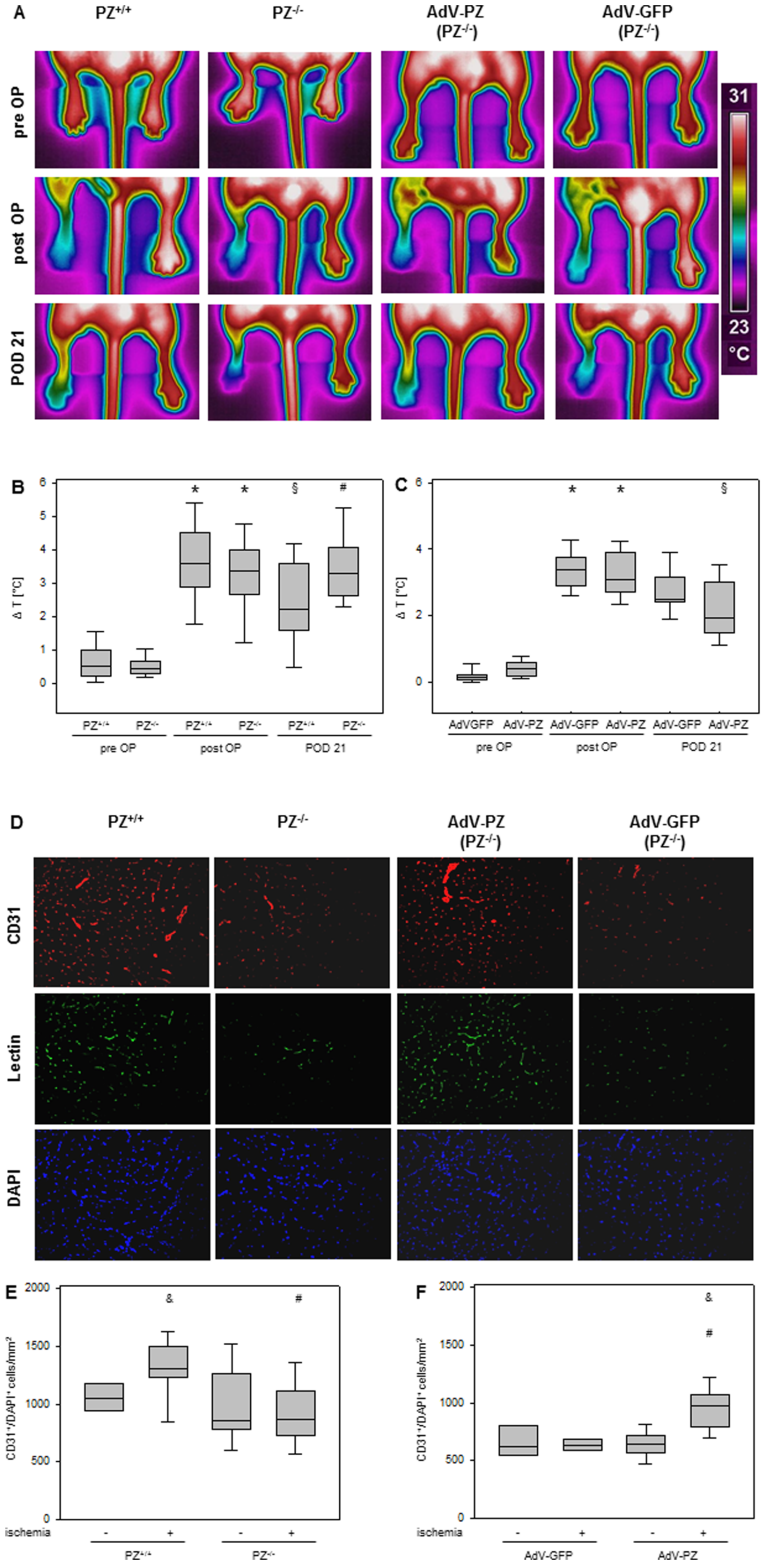
PZ in ischemia-induced neovascularization *in vivo*. **A**, Representative pictures of thermal imaging of mice hindlimbs. **B**, Quantitative summary of pad temperature differences. On POD 21 PZ^+/+^ mice showed a significant reduction in temperature difference compared to immediately after induction of ischemia and significant lower temperature difference to PZ^−/−^ mice, which offered no changes. Data are given as box plots indicating the median with the 25^th^ and 75^th^ percentiles. ANOVA on ranks repeated measures; * p<0.05 vs. pre OP, § p<0.05 vs. post OP, # p<0.05 vs. other genotype on POD 21, n = 11–16. **C**, After 21 days, PZ^−/−^ mice treated with AdV-PZ showed significant reduction in temperature difference in the ischemic hindlimb. Data are given as box plots indicating the median with the 25^th^ and 75^th^ percentiles. ANOVA on ranks repeated measures; * p<0.05 vs. pre OP, § p<0.05 vs. post OP; n = 10. **D**, Representative pictures of M. gastrocnemius muscle tissue after immunofluorescent staining for CD31 (red) or cell nuclei (DAPI, blue). Intracardiac injection of an endothelial specific lectin confirmed functional perfusion of CD31/DAPI double positive capillaries (green). **E**, PZ^+/+^ mice exhibited significant higher density of CD31/DAPI-double positive capillaries per square millimeter of ischemic M. gastrocnemius tissue compared to the non-ischemic PZ^+/+^ muscle tissue. In muscle tissue of PZ^−/−^ mice, ischemia did not result in remarkable neovascularization. The number of CD31/DAPI double-positive cells in ischemic PZ^−/−^ mice M. gastrocnemius was even significantly lower compared to ischemic PZ^+/+^ mice. Data are given as box plots indicating the median with the 25^th^ and 75^th^ percentiles. ANOVA on ranks; & p<0.05 vs. non-ischemic, # p<0.05 vs. other genotype with ischemia; n = 11–16. **F**, After exposure of PZ^−/−^ mice to AdV-PZ, the compromised angiogenic phenotype of PZ^−/−^ mice after induction of ischemia was significantly reversed. Data are given as box plots indicating the median with the 25^th^ and 75^th^ percentiles. ANOVA on ranks; & p<0.05 vs. non-ischemic, # p<0.05 vs. AdV-GFP with ischemia, n = 10.

Based on the pro-angiogenic properties of PZ *in vitro*, we evaluated the relevance of these findings *in vivo* by using the murine model of hindlimb ischemia. Thermography served for indirect assessment of hindlimb perfusion by calculating temperature differences between the non-operated and operated extremity (Figure 2 A). Quantification of temperature differences between the hindlimbs revealed a significant increase in both PZ^+/+^ and PZ^−/−^ mice after preparation of hindlimb ischemia compared to the pre-operation conditions (Figure 2 B). In PZ^+/+^ mice temperature difference between mouse pads decreased significantly on POD 21, indicating enhanced reperfusion in the ischemic hindlimb. Of note, temperature difference between hindlimbs of PZ^−/−^ mice remained at a level comparable to that found directly after the induction of ischemia (p<0.05 vs. PZ^+/+^ mice on POD 21). At POD 21 neovascularization was evaluated by enumeration of CD31/DAPI double positive capillaries in the M. gastrocnemius. As shown in Figure 2 D and E, induction of ischemia in PZ^+/+^ mice resulted in an increased capillary density, while PZ^−/−^ mice did not exhibit increased angiogenic activity in response to ischemia (p<0.05 vs. PZ^+/+^ mice).

### PZ expressing adenovirus reverses the diminished angiogenic phenotype of PZ^−/−^ mice

In order to analyse whether reduced angiogenesis in PZ^−/−^ mice could indeed be ascribed to the deficiency for PZ and is not related to other features of this genetic phenotype, PZ^−/−^ mice were administered a PZ expressing adenovirus (AdV-PZ) resulting in physiological PZ-plasma concentrations with a median of 0.8 µg/ml at a range of 0.5–1.0 µg/ml (25^th^ and 75^th^ percentiles) on POD 21. Quantification of thermal images revealed a significant reduction in temperature difference on POD 21 in PZ^−/−^ mice exposed to AdV-PZ, indicating improved blood perfusion in the ischemic hindlimb by reversing the phenotype of PZ^−/−^ mice (Figure 2 C). Analysis of the number of CD31/DAPI double positive capillaries in M. gastrocnemius tissue revealed higher numbers in PZ^−/−^ mice following application of AdV-PZ compared to their non-ischemic hindlimb and also compared to mice receiving AdV-GFP (serving as control virus) only, displaying improved neovascularization by substitution of PZ^−/−^ mice with PZ (Figure 2 D and F).

### Targeting PZ and CXCR4 with siRNA results in decreased migratory capacity and endothelial tube formation of HUVECs *in vitro*

**Figure 3:**
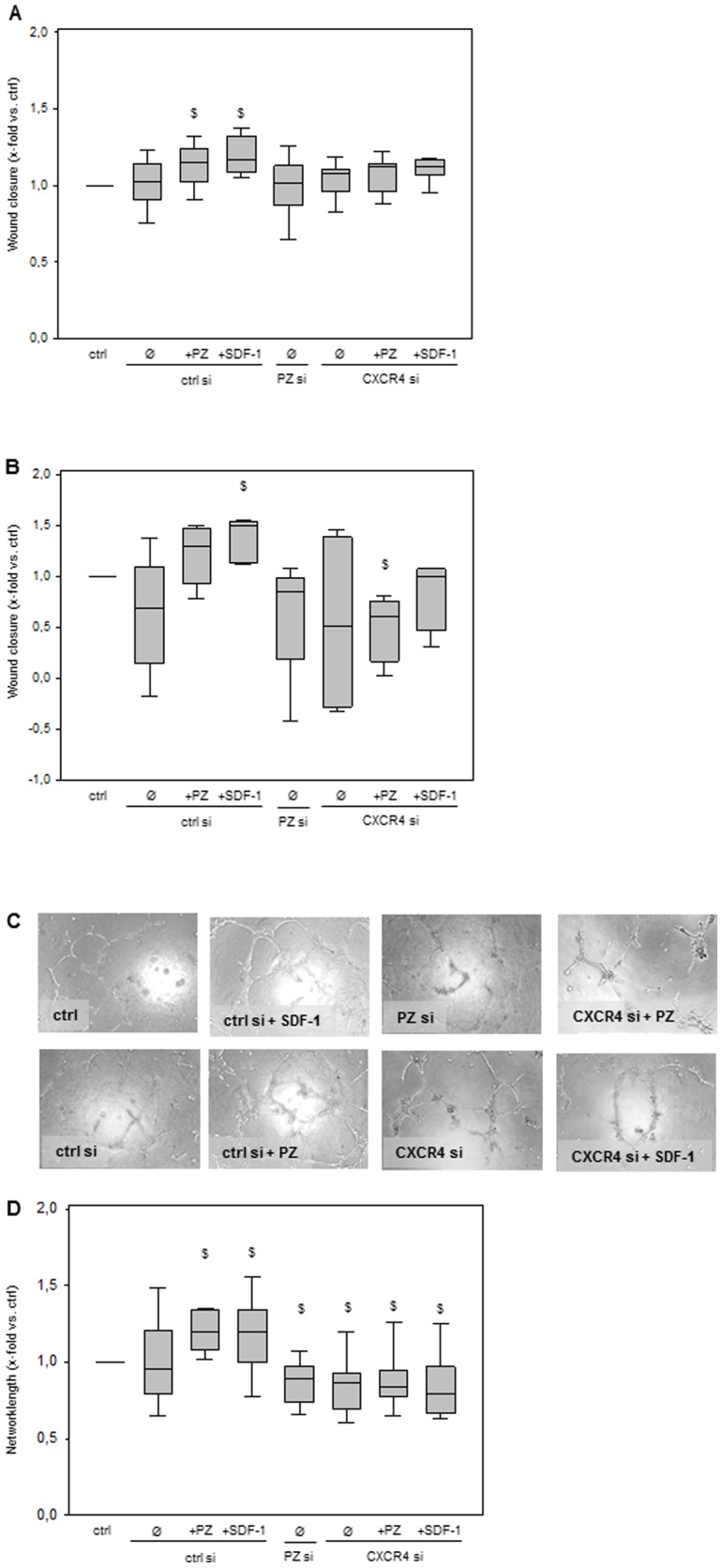
Targeting PZ and CXCR4 with siRNA results in decreased migratory capacity and endothelial tube formation *in vitro*. PZ and CXCR4 knock-down in unstimulated HUVECs as well as CXCR4 knock-down in PZ and SDF-1 stimulated HUVECs resulted in migration capacity comparable to that of ctrl after both 8 (**A**) and 24 hours (**B**). Data are given as box plots indicating the median with the 25^th^ and 75^th^ percentiles. Ø corresponds to unstimulated cells. ANOVA on ranks; $ p<0.05 vs. ctrl; n = 4 independent experiments. Representative images (**C**) and quantitative analysis (**D**) of capillary-like tube formation. HUVECs targeted with siRNA against PZ or CXCR4 showed significantly reduced capacity for capillary-like tube formation compared to ctrl. Addition of PZ or SDF-1 to CXCR4 siRNA targeted cells failed to restore endothelial tube formation. Data are given as box plots indicating the median with the 25^th^ and 75^th^ percentiles. Ø corresponds to unstimulated cells. ANOVA on ranks; $ p<0.05 vs. ctrl; n = 4 independent experiments.

To strengthen the findings of pro-angiogenic action of PZ *in vitro* knock-down of PZ and CXCR4 was used to verify the contribution of PZ to basic angiogenic processes. The migration assay was performed after knock-down of PZ and CXCR4 by siRNA targeting (Figure 3 A and B). PZ knock-down in HUVECs reduced the migratory capacity slightly below control values after 8 and 24 hours. In addition, CXCR4 knock-down resulted in unstimulated as well as PZ and SDF-1 exposed cells in migration capacity comparable to that of unstimulated cells at both time-points (Figure 3 A and B). Performing matrigel angiogenesis assay with PZ siRNA targeted endothelial cells revealed a significantly reduced capacity in formation of tubular-like structures (Figure 3 C and D). Comparably, siCXCR4-targeted cells did not show increased tube formation and did not respond to stimulation with either PZ or SDF-1 (Figure 3 C and D).

### PZ stimulates endothelial CXCR4 surface expression

**Figure 4:**
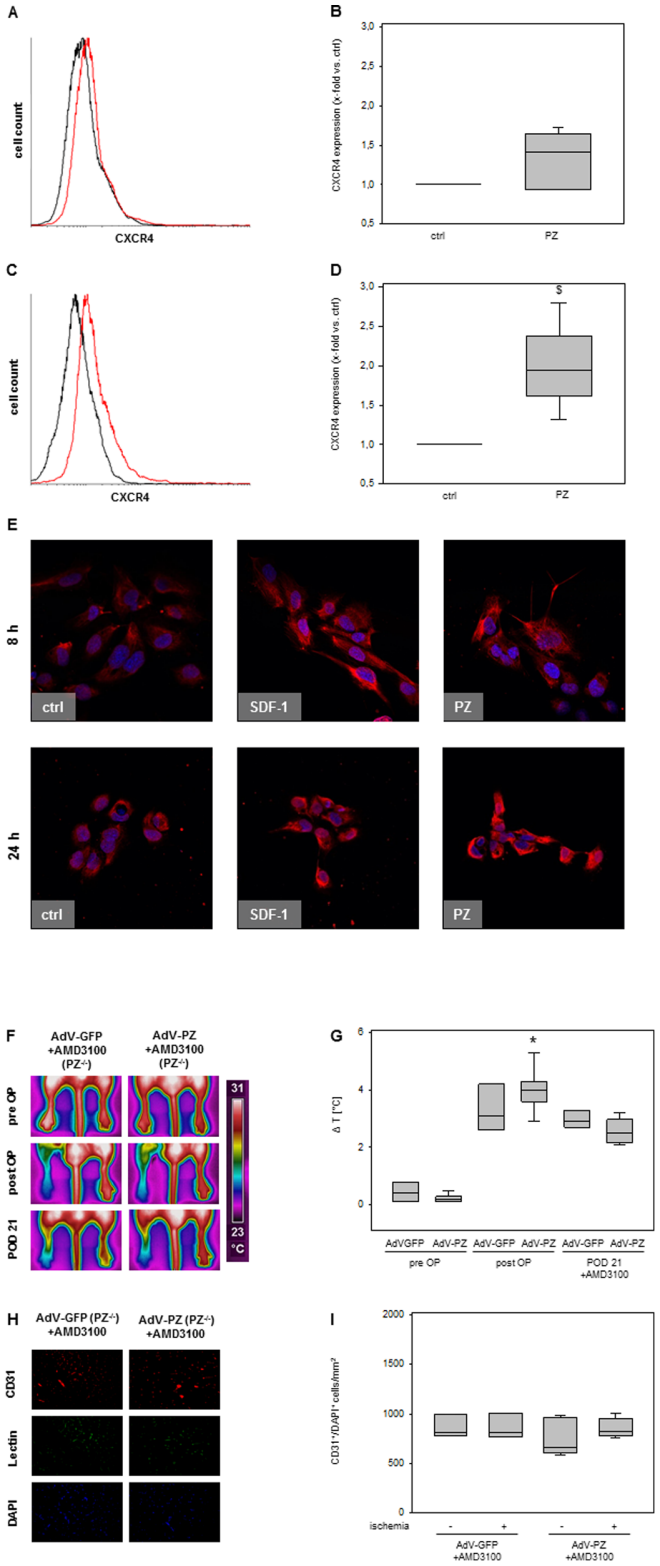
PZ upregulates CXCR4 surface expression on endothelial cells *in vitro* and mediates its angiogenic effects via CXCR4 *in vivo*. Flow cytometric analysis of CXCR4 surface expression on HUVECs after 8 and 24 hours of incubation with PZ (red line), displayed in representative histograms (**A** and **C**). Quantitative analysis (**B** and **D**) of CXCR4 expression. Stimulation with PZ resulted in an 1.4-fold (B) and almost 2-fold (D) increase of CXCR4 expression vs. unstimulated cells (ctrl, black line). Data are given as box plots indicating the median with the 25^th^ and 75^th^ percentiles. ANOVA on ranks; p<0.05; $ vs. ctrl; n = 4–8 independent experiments. ANOVA on ranks; p<0.05; $ vs. ctrl; n = 4–8 independent experiments. **E**, Representative images of confocal laser scanning microscopy of HUVECs stimulated for 8 or 24 hours with SDF-1 (50 ng/ml) or PZ (3 µg/ml), displaying an increased surface expression of CXCR4 after stimulation with both substances. **F**, Representative pictures of thermal imaging of mice hindlimbs. **G**, Quantitative summary of pad temperature differences pre OP, post OP and on POD 21. In both groups treated with AMD3100 no significant change in temperature difference on POD 21 was detectable. Data are given as box plots indicating the median with the 25^th^ and 75^th^ percentiles. ANOVA on ranks repeated measures; * p<0.05 vs. pre OP; n = 3–6. **H**, Representative pictures of M. gastrocnemius after immunofluorescent staining for CD31 (red) or cell nuclei (DAPI, blue). **I**, Quantitative summary of enumbered CD31/DAPI double positive cells revealed no significant increase after induction of ischemia in both groups. Data are given as box plots indicating the median with the 25^th^ and 75^th^ percentiles. ANOVA on ranks; n = 3–6.

*In vitro* effects of PZ on endothelial cells were comparable to those exerted by SDF-1, which, together with its canonical receptor CXCR4, plays a central role in mediating hypoxia-driven neovascularization, by inducing a G-protein-coupled downstream signaling [25], [26]. To further prove the hypothesis, whether CXCR4 could be required for mediating the pro-angiogenic effects of PZ, the functional response of endothelial cells in the matrigel assay was investigated after G-protein inhibition by incubation of endothelial cells with PZ in combination with 100 ng/ml pertussis toxin (PTX). Combined incubation with PZ and PTX abolished the pro-angiogenic effect of PZ significantly, compared to PZ only treated endothelial cells (Figure 1 D and E), while adding PTX alone to endothelial cells had no effect on endothelial tube formation. Stimulation of HUVECs with PZ for 8 and 24 hours resulted in an 1.4-fold and almost 2-fold increase of CXCR4 surface expression vs. unstimulated ctrl (Figure 4 A–D), as detected by flow cytometric analysis. Increased expression of CXCR4 is also displayed in representative confocal laser scanning microscopic images of 8 and 24 hours PZ and SDF-1 stimulated HUVECs as well as unstimulated ctrl, as shown in Figure 4 E.

### Application of AMD3100 to AdV-PZ exposed PZ^−/−^ mice reverses the beneficial effects of AdV-PZ

To evaluate if the surface expression of CXCR4 by PZ on stimulated HUVECs is of relevance *in vivo*, PZ^−/−^ mice were exposed to AdV-PZ or AdV-GFP and received three single injections of AMD3100. On POD 21, neither AMD3100-treated PZ^−/−^ mice in the AdV-PZ nor in the AdV-GFP treatment group offered remarkable improvement in reperfusion of the ischemic hindlimb (Figure 4 F and G). Correspondingly, there was no increase in CD31/DAPI double positive cells in M. gastrocnemius tissue in both groups above the level detected in non-ischemic hindlimbs (Figure 4 H and I). In this regard, application of AMD3100 suspended the beneficial pro-angiogenic effect of AdV-PZ in PZ^−/−^ mice, indicating that the restoration of the PZ^+/+^ phenotype by AdV-PZ in PZ^−/−^ mice is CXCR4-linked.

## Discussion

Despite its characterization in human plasma in 1984 [2], the physiological function of PZ is not yet completely understood. In line with the prothrombotic phenotype of PZ deficiency observed in mice [7], [8] the meta-analytical study of Sofi et al. [27] demonstrated that low levels of PZ were associated with an increased risk for arterial thrombosis, pregnancy complications and venous thromboembolic diseases. Overall, patients with low levels of PZ showed nearly a three-fold increased risk of thrombotic events [27]. Beyond the contribution of PZ in coagulation, there are several clinical studies which show an association between low PZ levels and the occurrence of coronary and peripheral atherosclerosis [28], [29], [11], [12] signifying a basic role of PZ in vascular biology, including angiogenesis. There is a clear link between hypoxia-driven angiogenesis and coagulation. By activating the hypoxia-inducible transcription factor HIF-1α, thrombin triggers the expression of several angiogenic molecules and activated platelets release large stores of angiogenic factors, such as vascular endothelial growth factor (VEGF), platelet-derived growth factor (PDGF), transforming growth factor-β (TGF-β), and interleukin-6 (IL-6) [30]. Thus, when coagulation is initiated, a cascade of angiogenic signals is also generated. Together, coagulation and angiogenesis could cooperate in stabilizing new blood vessels, repairing injured vessels, and stimulating the sprouting of new vessels [31].

The purpose of the present study was to unravel the role of PZ in angiogenesis by means of established *in vitro* and *in vivo* assays. Herein, we communicate the following major findings: (i) *In vitro*, PZ stimulated migratory activity and capillary-like tube formation of endothelial cells comparable to SDF-1, implying a pro-angiogenic potential. (ii) Accordingly, PZ^−/−^ mice exhibited diminished hypoxia-driven neovascularis zation and reperfusion in post-ischemic hindlimbs, (iii) which was restored by adenoviral gene transfer up to levels seen in PZ^+/+^ mice. (iv) The increased surface expression of CXCR4 on PZ-stimulated endothelial cells and the abrogated restoration of PZ^−/−^ mice via AdV-PZ after concomitant treatment with the CXCR4 antagonist AMD3100 supported the idea that PZ mediates angiogenesis via a G-protein coupled pathway and involves the SDF-1/CXCR4 axis. (v) This is underlined by the fact that addition of the G-protein inhibitor PTX to PZ-stimulated endothelial cells abolished the effect of PZ on capillary-like tube formation. (vi) Using siRNA-targeting for knock-down of PZ in *in vitro* assays resulted in reduced endothelial tube formation. Furthermore, this addition of PZ to endothelial cells targeted with siCXCR4 failed to restore the capacity for tube formation.

PZ increased the formation of capillary tube-like structures from endothelial cells *in vitro*, comparably as this has been shown for aPC, a serine protease with central role in physiological coagulation [32]. aPC mediates this effect by binding to its endothelial protein C receptor (EPCR) and activating protease activated receptor (PAR-1) [18]. With respect to the structural homology of PZ to aPC, comparable mechanisms might underlie the action profile of PZ, though not studied yet in full detail. However, comparably to aPC, which has been shown to be synthesized in HUVECs [16], endothelial cells also synthesize PZ [20].

The reduced capacity of endothelial cells to migrate and form tubular structures after knock-down of PZ *in vitro* is consistent with the reduced angiogenic response in PZ^−/−^ mice employing the hindlimb ischemia model. In line with this, the reconstitution of physiological plasma concentration of PZ by adenoviral gene transfer in PZ^−/−^ mice, which restored the capability of mice to adequately respond to hindlimb ischemia with neovascularization, underlines an involvement of PZ in angiogenic processes.

Even though a possible down-stream signalling effect of PZ remains unknown, the immunolocalization of PZ in the endothelium of arterial and venous vessel sections implies the binding of PZ to a postulated, but so far not identified endothelial receptor [20], which could be the crucial point in PZ-mediated effects. Addition of PTX to PZ stimulated cells reverted the pro-angiogenic effect of PZ significantly, which implies that PZ mediates its action via a G-protein coupled pathway. Antagonizing CXCR4 by AMD3100 resulted in an insufficient restoration of vascularization in AdV-PZ-pretreated PZ^−/−^ mice. This observation and the flow cytometric data of the PZ-induced increase of CXCR4 surface expression on endothelial cells allow to assume that PZ affects CXCR4 surface expression via a G-protein coupled pathway. However, PZ only stimulates CXCR4 surface expression and seems not to be a ligand of CXCR4 like SDF-1, as PZ stimulation did not activate the CXCR4-associated signal cascade, namely pERK and pAKT (data not shown).

In conclusion, our results confirm the importance of PZ in vascular homeostasis and extend the current knowledge by demonstrating a relevant contribution of PZ in ischemia-induced angiogenesis. The mode of PZ action with the underlying molecular mechanisms remains to be clarified. Nevertheless, PZ might offer future potential as a novel approach for therapeutic angiogenesis.

## Supporting Information

Figure S1 **Genotyping of PZ mice.** All animals were genotyped for presence or absence of PZ by PCR using genomic DNA isolated from the tail tip (aqua dest. served as negative control; M, marker).
(TIF)

Figure S2 **Kinetics of PZ expressing adenovirus** ***in vitro*** **.** **A**, Representative pictures of phase contrast microscopy of H1299 cells infected with PZ expressing adenovirus (AdV-PZ), only GFP expressing adenovirus (AdV-GFP) or non infected cells (Mock). 100× magnification **B**, Representative fluorescence microscopy pictures of H1299 cells infected with PZ expressing adenovirus (AdV-PZ), only GFP expressing adenovirus (AdV-GFP) or non-infected cells (Mock). Original magnification ×100. **C**, Representative Western Blot of H1299 cells exposed to AdV-PZ or AdV-GFP or non-infected cells (Mock) displaying a band at 62 kDa only in cells infected with AdV-PZ. β-actin served as loading control. **D**, PZ concentrations in the supernatant of H1299 cells measured by ELISA, data are given in mean ± SEM; n = 3 independent experiments; n.d., not detectable.
(TIF)

Figure S3 **Kinetic of PZ expressing adenovirus** ***in vivo*** **.** **A**, Representative intravital fluorescence microscopy images of liver displaying GFP-fluorescent hepatocytes of PZ^−/−^ mice exposed to AdV-PZ or AdV-GFP over a period of 96 hours. Original magnification ×50. **B**, Representative immuno-histochemical images of hepatic tissue stained for GFP in PZ^−/−^ mice exposed to AdV-PZ or AdV-GFP. Original magnification ×100. **C**, Representative immunohistochemical images of hepatic tissue stained for PZ in PZ^−/−^ mice exposed to AdV-PZ or AdV-GFP. Original magnification ×100. **D**, PZ plasma concentrations measured by ELISA in PZ^−/−^ mice exposed to AdV-PZ or AdV-GFP; Data are given in mean ± SEM; n = 3.
(TIF)

Material and Methods S1 **Further information about genotyping of PZ mice, adenovirus vector production and adenovirus kinetic studies can be found in the supporting information.**
(DOCX)
