# Supplementary figures and images for: Protein Z Exerts Pro-Angiogenic Effects and Upregulates CXCR4
Source: PLoS One. 2014 Dec 4;9(12):e113554. doi: 10.1371/journal.pone.0113554 (PMC4256373; doi:10.1371/journal.pone.0113554)

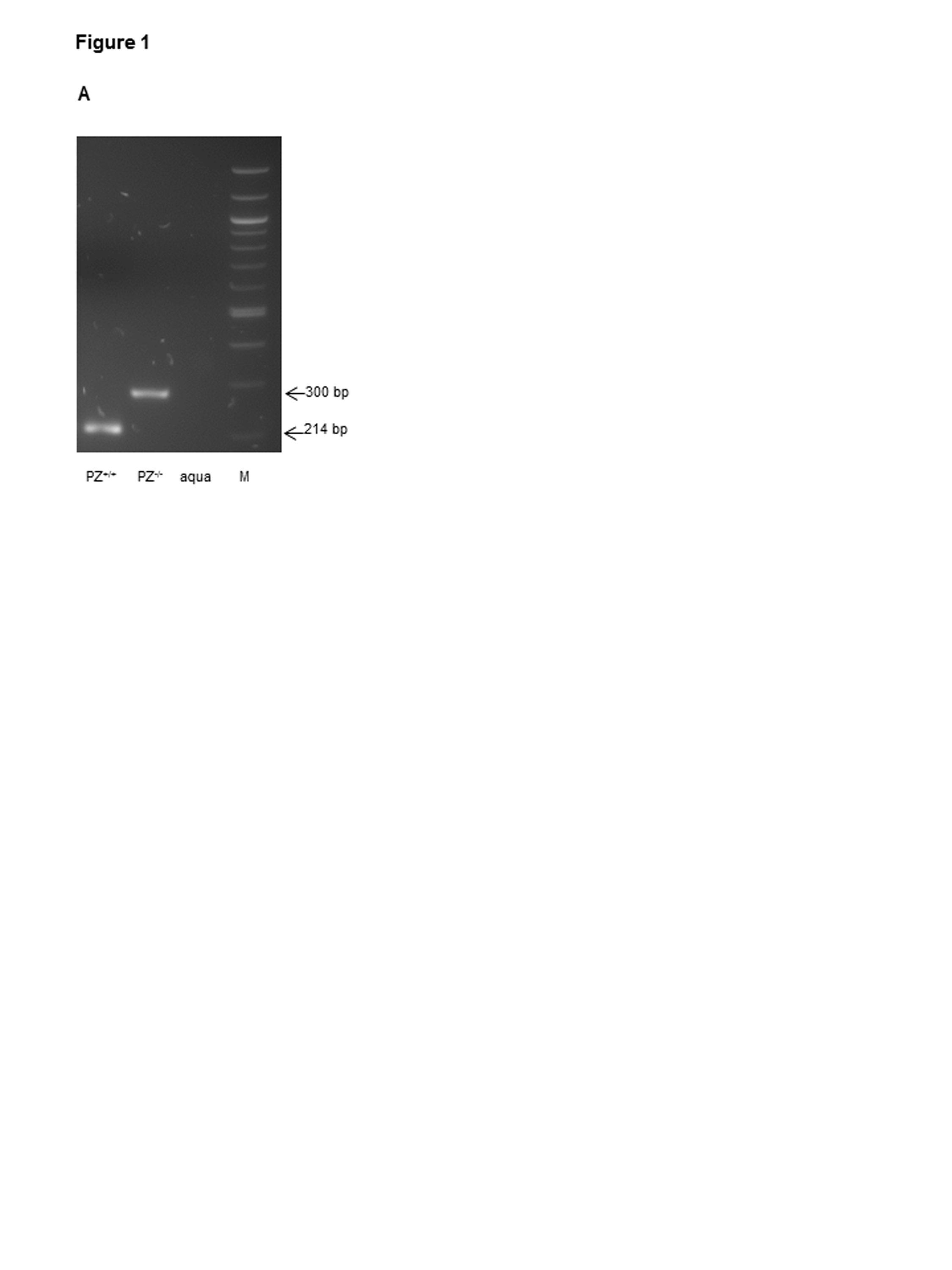

Supplement: Figure S1 — Genotyping of PZ mice. All animals were genotyped for presence or absence of PZ by PCR using genomic DNA isolated from the tail tip (aqua dest. served as negative control; M, marker). (TIF) [file pone.0113554.s001.tif]

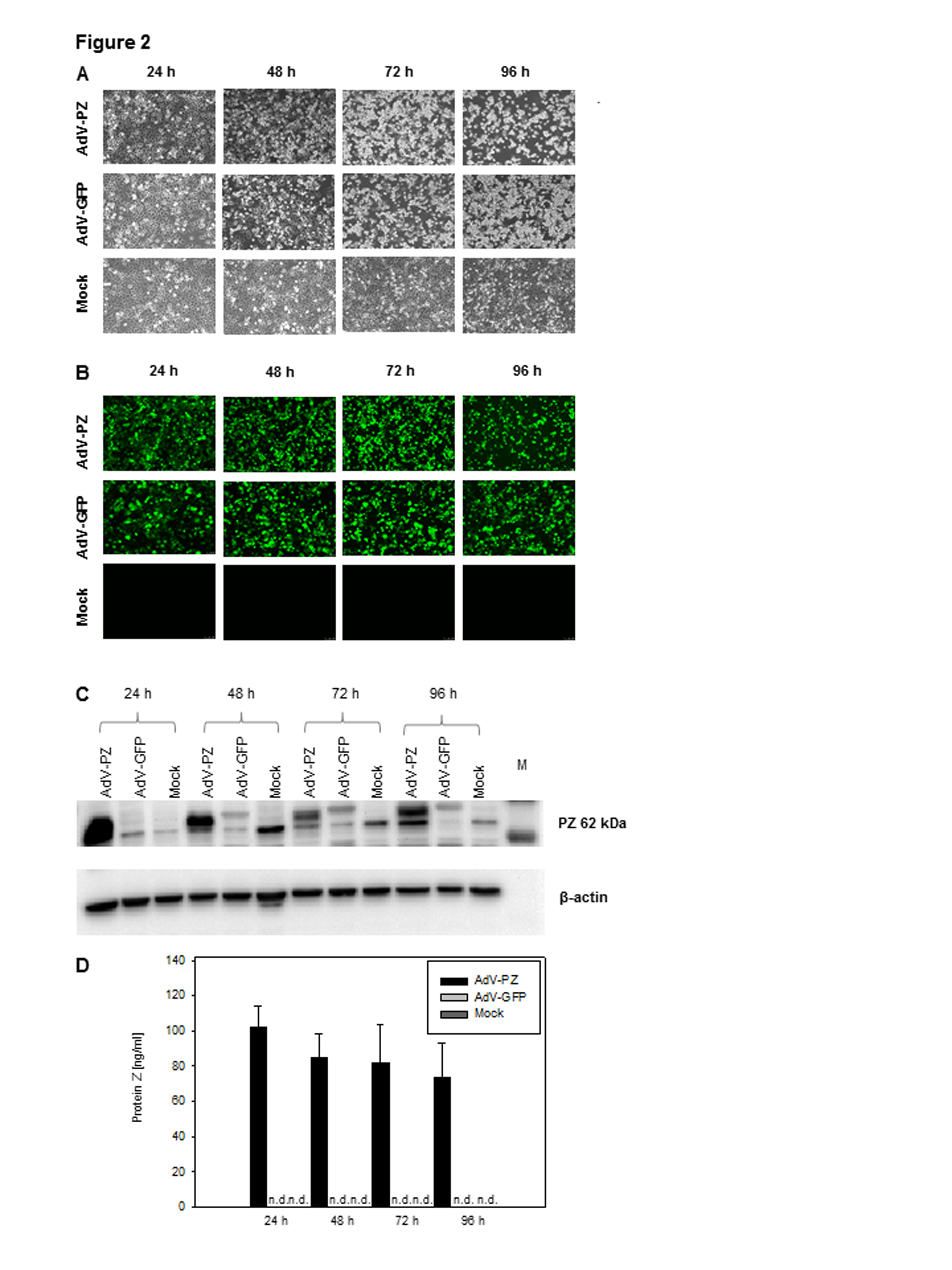

Supplement: Figure S2 — Kinetics of PZ expressing adenovirus in vitro . A, Representative pictures of phase contrast microscopy of H1299 cells infected with PZ expressing adenovirus (AdV-PZ), only GFP expressing adenovirus (AdV-GFP) or non infected cells (Mock). 100× magnification B, Representative fluorescence microscopy pictures of H1299 cells infected with PZ expressing adenovirus (AdV-PZ), only GFP expressing adenovirus (AdV-GFP) or non-infected cells (Mock). Original magnification ×100. C, Representative Western Blot of H1299 cells exposed to AdV-PZ or AdV-GFP or non-infected cells (Mock) displaying a band at 62 kDa only in cells infected with AdV-PZ. β-actin served as loading control. D, PZ concentrations in the supernatant of H1299 cells measured by ELISA, data are given in mean ± SEM; n = 3 independent experiments; n.d., not detectable. (TIF) [file pone.0113554.s002.tif]

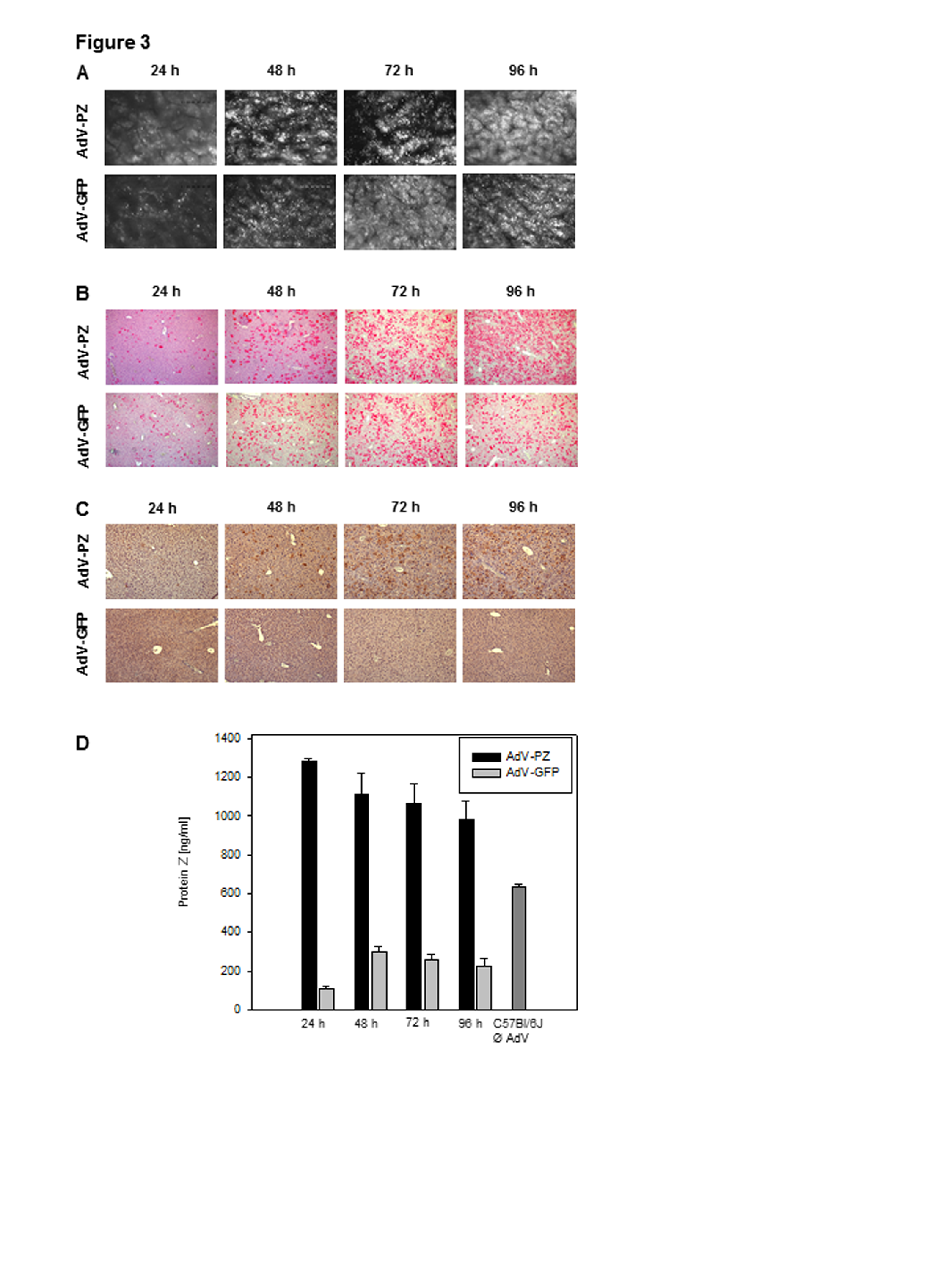

Supplement: Figure S3 — Kinetic of PZ expressing adenovirus in vivo . A, Representative intravital fluorescence microscopy images of liver displaying GFP-fluorescent hepatocytes of PZ−/− mice exposed to AdV-PZ or AdV-GFP over a period of 96 hours. Original magnification ×50. B, Representative immuno-histochemical images of hepatic tissue stained for GFP in PZ−/− mice exposed to AdV-PZ or AdV-GFP. Original magnification ×100. C, Representative immunohistochemical images of hepatic tissue stained for PZ in PZ−/− mice exposed to AdV-PZ or AdV-GFP. Original magnification ×100. D, PZ plasma concentrations measured by ELISA in PZ−/− mice exposed to AdV-PZ or AdV-GFP; Data are given in mean ± SEM; n = 3. (TIF) [file pone.0113554.s003.tif]
