# Supplementary material for: Protein Z Exerts Pro-Angiogenic Effects and Upregulates CXCR4
Source: PLoS One. 2014 Dec 4;9(12):e113554. doi: 10.1371/journal.pone.0113554 (PMC4256373; doi:10.1371/journal.pone.0113554)
Supplement: Material and Methods S1 — Further information about genotyping of PZ mice, adenovirus vector production and adenovirus kinetic studies can be found in the supporting information. (DOCX) [file pone.0113554.s004.docx]

**Supporting Informations**

**Material and Methods**

**Genotyping of Protein Z mice.** All animals were genotyped for presence or absence of Protein Z (PZ) by PCR, as described by Yin et al. [1] and Zhang et al. [2] using genomic DNA isolated from the tail tip. Genomic DNA was prepared by using NaOH and heating the probes for 1 h at 97°C and subsequently adjusting TrisHCl at a pH of 8. PCR was performed using peqGOLD Taq-DNA-Polymerase (PEQLAB Biotechnologie GmbH, Erlangen, Germany) and a Eppendorf Mastercycler gradient (Eppendorf AG, Hamburg, Germany). A representative image displaying amplification products in a PCR gel of genotyped PZ mice is given in Supplemental Figure 1.

**Adenovirus vector production.** Ad serotype 5-derived wild-type vector (AdV-GFP), expressing the green fluorescence protein (GFP) and murine PZ-expressing vector (AdV-PZ), were generated by homologous recombination following cotransfection of a PZ coding plasmid (plasmid: 1018398_murine_Proz_pAdTrack-CMV_F119; imaGenes GmbH, Berlin, Germany) or pADTrack-CMV with pAdEasy1 in E. coli BJ5183. Ad vectors were propagated in HEK293 cells, purified by CsCl buoyant density centrifugation, and measured at OD260.

**Adenovirus vector kinetic *in vitro*.** *In vitro* adenoviral infections were carried out at a multiplicity of infection (MOI) that allows a proper transduction of H1299 cells (human non-small lung carcinoma cell line, ATCC®, USA), an immortalized cell line which does not produce PZ per se (MOI = 10, see Figure 2D) and expresses the coxsackie and adenovirus receptor [3]. H1299 cells were grown in 10 cm dishes at a density of 1*10^6^ cells and grown at 37°C in a 5% CO_2_ humidified atmosphere. After 24 hours in culture, cells were infected with AdV-PZ, AdV-GFP or were not infected (control=Mock). The successful genetransfer was verified optically by GFP exposure of the cells with an inverse fluorescence microscope every 24 hours after infection (DMI 4000 B, Leica, Germany). Supernatant was harvested and concentrated by ultrafiltration with Amicon® Ultra-15 Centrifugal Filter Devices (Merck Millipore, Tullagreen, Ireland) up to a volume <300 µl. PZ was determined in the supernatants using an ELISA (Hyphen BioMed, France) as shown in Supplemental Figure II D. Cells were also harvested for analysis by Western Blotting. PZ absence (AdV-GFP and Mock) or presence (AdV-PZ) was detected using an anti-PZ antibody from abcam (Ms mAb, ab86372) (see Supplemental Figure 2 C).

**Adenovirus vector kinetic *in vivo*.** For analysis of efficacy and kinetic of AdV-PZ *in vivo* PZ^-/-^ mice were anesthetized (for details see section Material and Methods) and placed on a warming pad. The jugular vein was exposed carefully with forceps and the virus suspension (1*10^8^ particles in saline with a final volume of 50 µl) was injected slowly. Every 24 hours until 96 hours after injection intravital fluorescence microscopic documentation of GFP-fluorescence was performed in the left liver lobe covered with a glass slide (see Supplemental Figure III A), using a Zeiss fluorescence microscope equipped with a 100W mercury lamp and a filter set for blue epi-illumination (Axiotech Vario; Zeiss, Jena, Germany) as described by Abshagen et al. [4]. All experiments were approved by the local government (LALLF M-V 7221.3-1.1-072/11). After the microscopy, liver tissue was sampled for subsequent histological and immunhistochemical analysis and blood was sampled for detection of PZ by using an ELISA (Hyphen BioMed, France). Liver tissue was fixed in 4% phosphate buffered formalin and embedded in paraffin to cut 5 µm sections, which were immunhistochemically stained for GFP (ab6556, abcam) and PZ (sc-23505, Santa Cruz) (see Supplemental Figure 3B and C).

**References**

1. Yin ZF, Huang ZF, Cui J, Fiehler R, Lasky N, Ginsburg D, Broze GJ Jr (2000) Prothrombotic phenotype of protein Z deficiency. Proc Natl Acad Sci U S A 97: 6734-6738.
2. Zhang J, Tu Y, Lu L, Lasky N, Broze GJ Jr (2008) Protein Z-dependent protease inhibitor deficiency produces a more severe murine phenotype than protein Z deficiency. Blood 111: 4973-4978.
3. Wu PC, Wang Q, Dong ZM, Chu E, Roberson RS, Ivanova IC, Wu DY (2010) Expression of coxsackie and adenovirus receptor distinguishes transitional cancer states in therapy-induced cellular senescence. Cell Death Dis. 1: e70.
4. [Abshagen K](http://www.ncbi.nlm.nih.gov/pubmed?term=Abshagen%20K%5BAuthor%5D&cauthor=true&cauthor_uid=16500681), [Eipel C](http://www.ncbi.nlm.nih.gov/pubmed?term=Eipel%20C%5BAuthor%5D&cauthor=true&cauthor_uid=16500681), [Menger MD](http://www.ncbi.nlm.nih.gov/pubmed?term=Menger%20MD%5BAuthor%5D&cauthor=true&cauthor_uid=16500681), [Vollmar B](http://www.ncbi.nlm.nih.gov/pubmed?term=Vollmar%20B%5BAuthor%5D&cauthor=true&cauthor_uid=16500681) (2006) Comprehensive analysis of the regenerating mouse liver: an in vivo fluorescence microscopic and immunohistological study. [J Surg Res.](http://www.ncbi.nlm.nih.gov/pubmed/?term=abshagen+2006) 134: 354-62.

**Supporting Information Figure Legends**

**Figure 1. Genotyping of PZ mice.** All animals were genotyped for presence or absence of PZ by PCR using genomic DNA isolated from the tail tip (aqua dest. served as negative control; M, marker).

**Figure 2. Kinetic of PZ expressing adenovirus *in vitro*. A**, Representative pictures of phase contrast microscopy of H1299 cells infected with PZ expressing adenovirus (AdV-PZ), only GFP expressing adenovirus (AdV-GFP) or not infected (Mock). 100x magnification **B**, Representative fluorescence microscopy pictures of H1299 cells infected with PZ expressing adenovirus (AdV-PZ), only GFP expressing adenovirus (AdV-GFP) or not infected (Mock). Original magnification x100. **C**, Representative Western Blot of H1299 cells exposed to AdV-PZ or AdV-GFP or non-infected cells (Mock) displaying a band at 62 kDa only in cells infected with AdV-PZ. β-actin served as loading control. **D**, PZ concentrations in the supernatant of H1299 cells measured by ELISA, data are given in mean ± SEM, n=3 independent experiments; n.d., not detectable.

**Figure 3.** **Kinetic of PZ expressing adenovirus *in vivo***. **A**, Representative intravital fluorescence microscopy images of liver displaying GFP-fluorescent hepatocytes of PZ^-/-^ mice exposed to AdV-PZ or AdV-GFP over a period of 96 hours. Original magnification x50. **B**, Representative immuno-histochemical images of hepatic tissue stained for GFP in PZ^-/-^ mice exposed to AdV-PZ or AdV-GFP. Original magnification x100. **C**, Representative immunohistochemical images of hepatic tissue stained for PZ in PZ^-/-^ mice exposed to AdV-PZ or AdV-GFP. Original magnification x100. **D**, PZ plasma concentrations measured by ELISA in PZ^-/-^ mice exposed to AdV-PZ or AdV-GFP; Data are given in mean ± SEM, n=3.
